# The effect of different posterior inclinations of tibial component on tibiofemoral contact pressures after unicompartmental knee arthroplasty

**DOI:** 10.1186/s13018-023-04222-5

**Published:** 2023-11-29

**Authors:** Bo Yuan, Zhongjun Mo, Kuan Zhang, Xu Zhu, Songhua Yan, Jizhou Zeng

**Affiliations:** 1 Department of Bone and Joint Surgery, Beijing Luhe Hospital, Capital Medical University, No. 82 Xinhua South Road, Tongzhou District, Beijing, 101149 China; 2 Beijing Key Laboratory of Rehabilitation Technical Aids for Old-Age Disability, Key Laboratory of Human Motion Analysis and Rehabilitation Technology of the Ministry of Civil Affairs, National Research Centre for Rehabilitation Technical Aids, Beijing, 100176 China; 3 Beijing Key Laboratory of Fundamental Research on Biomechanics in Clinical Application, No.10 Xitoutiao, You An Men Wai, Beijing, 100069 China; 4 School of Biomedical Engineering, Capital Medical University, Beijing, 100069 China

**Keywords:** Unicompartmental knee arthroplasty, Mobile-bearing, Posterior inclination of the tibial component, Finite element analysis, The contact pressures of tibiofemoral joint

## Abstract

**Background:**

Different posterior inclinations of tibial component after unicompartmental knee arthroplasty (UKA) may lead to different biomechanical characteristics of the knee joint. This finite element study was designed to investigate the tibiofemoral contact pressures after UKA with different posterior inclinations of tibial component.

**Methods:**

Finite element model of a healthy knee joint was constructed, and mobile-bearing (MB) UKA models with 5 different posterior inclinations (3°, 5°, 7°, 9° and 11°) of tibial components were simulated. The maximum contact pressures of tibial plateau cartilage in the lateral compartment and polyethylene insert in the medial compartment were calculated based on the ground reaction force and the angle of the knee flexion obtained by 3D motion capture system.

**Results:**

The loading ratio of medial and lateral compartments during standing stance (medial 54.49%, lateral 45.51%) and tibial anterior displacement (134 N, 3.89 mm) of healthy knee was basically consistent with previous experimental data. The maximum contact pressures of the medial meniscus and lateral tibial plateau cartilage of the healthy knee during standing stance were 2.14 MPa and 1.57 MPa, respectively. At the static standing phase, the maximum contact pressures of the polyethylene insert decreased from 17.90 to 17.29 Mpa, and the maximum contact pressures of the tibial plateau cartilage in the lateral compartment increased from 0.81 to 0.92 Mpa following an increase in the posterior inclination of the tibial component. At the first peak of ground reaction force, the maximum contact pressures of polyethylene insert increased from 22.37 to 25.16 MPa, and the maximum contact pressures of tibial plateau cartilage in the lateral compartment increased from 3.03 to 3.33 MPa, with the increase in the posterior inclination of the tibial component. At the second peak of ground reaction force, the maximum contact pressures of polyethylene insert decreased from 2.34 to 2.22 MPa with the increase in posterior inclination of tibial component.

**Conclusion:**

The preoperative and postoperative finite element models of MB UKA were well established. The results showed that the maximum contact pressures of the polyethylene insert did not change significantly with the increase in the posterior inclination of the tibial prosthesis, while the maximum contact pressures of the tibial plateau cartilage of the lateral compartment increased when the posterior inclination of the tibial prosthesis was > 7°. Our results also show that the maximum contact pressures were greater with an excessive inclination angle (11°) of the tibial component, and the pressures of the tibial plateau cartilage in the lateral compartment were more concentrated on the posterior area. This study, therefore, proposes that excessive osteotomy should be avoided.

## Introduction

Knee osteoarthritis (KOA) is a common and frequently occurring disease in the elderly [1], and most KOA patients suffer anterior medial osteoarthritis. At present, surgical treatment methods for anterior medial osteoarthritis are mainly divided into two categories: total knee arthroplasty (TKA) and unicompartmental knee arthroplasty (UKA). Compared with TKA, UKA is characterized by less trauma and quicker recovery periods [2]. As a kind of UKA surgery, MB UKA’s unique prosthesis design shares close similarity to the natural knee joint and can better restore the knee joint function. However, clinical outcomes have shown that the survival rate of MB UKA is significantly lower than that of TKA [3–5], mainly due to aseptic loosening and progressive development of lateral compartment osteoarthritis [6].

Contact stress is one of the important factors affecting the survival rate of MB UKA. The alteration of the alignment of the prosthesis, especially the posterior inclination of tibial prosthesis, leads to the change of contact pressures of the knee, which may cause aseptic loosening and concomitant osteoarthritis of MB UKA in the lateral compartment [7]. Excessive posterior inclination of tibial prosthesis may increase the probability of prosthesis loosening, the risk of anterior cruciate ligament injury and surgical revision [8].

Although different posterior inclinations of tibial prosthesis have different effects on knee function, scholars and clinicians still have greatly differing opinions on what is the best osteotomy angle should be. Weber et al. [9] suggested that a 4–8° posterior inclination of tibial prosthesis could help reduce the wearing of polyethylene insert. Aleto and Weber et al. [10, 11] believed that the posterior inclination of tibial prosthesis should be kept within the range of 0°–7° after surgery. Simpson et al. [12] found that the mean von Mises stress of the anteromedial tibia region did not change significantly with the increase in inclination of MB UKA. However, Small et al. [13] believed that the strain increased significantly in the posteromedial region of the proximal tibia when the tilt angle of tibial prosthesis increased from 5° to 10°. Therefore, it is of great significance to study the influence of posterior angle of tibial prosthesis on biomechanics of knee joint after MB UKA.

In this study, a finite element model of healthy knee joint was developed, and a MB UKA surgical simulation conducted to establish the influence of the maximum contact pressures on the polyethylene insert and the lateral tibial plateau cartilage surface after subjecting the knee to different posterior inclinations of tibial prosthesis. These pressures were calculated based on the joint force and motion characteristics of the gait cycle.

## Methods

### Modeling of healthy and MB UKA knee joint

A healthy female (48 years old, 165 cm height, 65 kg weight) with no history of knee degeneration or trauma as confirmed by X-ray and magnetic resonance imaging (MRI) was recruited in the study. Following a signed informed consent for imaging, 256-slice spiral CT (Philips, Brilliance iCT) was performed for full length CT scan of the left lower limb, ranging from hip- to ankle joint. The scanning layer thickness was 1 mm; A 3.0 T magnetic resonance scanner (Combined image UI770) was used to perform sagittal MR scan at the left knee joint with a thickness of 1 mm.

Mimics 20.0 (Materialise Ltd., Leuven, Belgium) was used to extract 3D models of bone, cartilage, meniscus, anterior and posterior cruciate ligaments, medial and lateral collateral ligaments from CT and MR images. Rapidform 2006 reverse engineering software (INUS Technology, Inc., Seoul, South Korea) was used to materialize the 3D model of each structure. Abaqus 6.14–2 finite element analysis software (Dassault Systemes SimuliaCoip., Providence, RI, USA) was imported for assembly and registration, and the 3D solid model of healthy knee joint was developed.

**Fig. 1:**
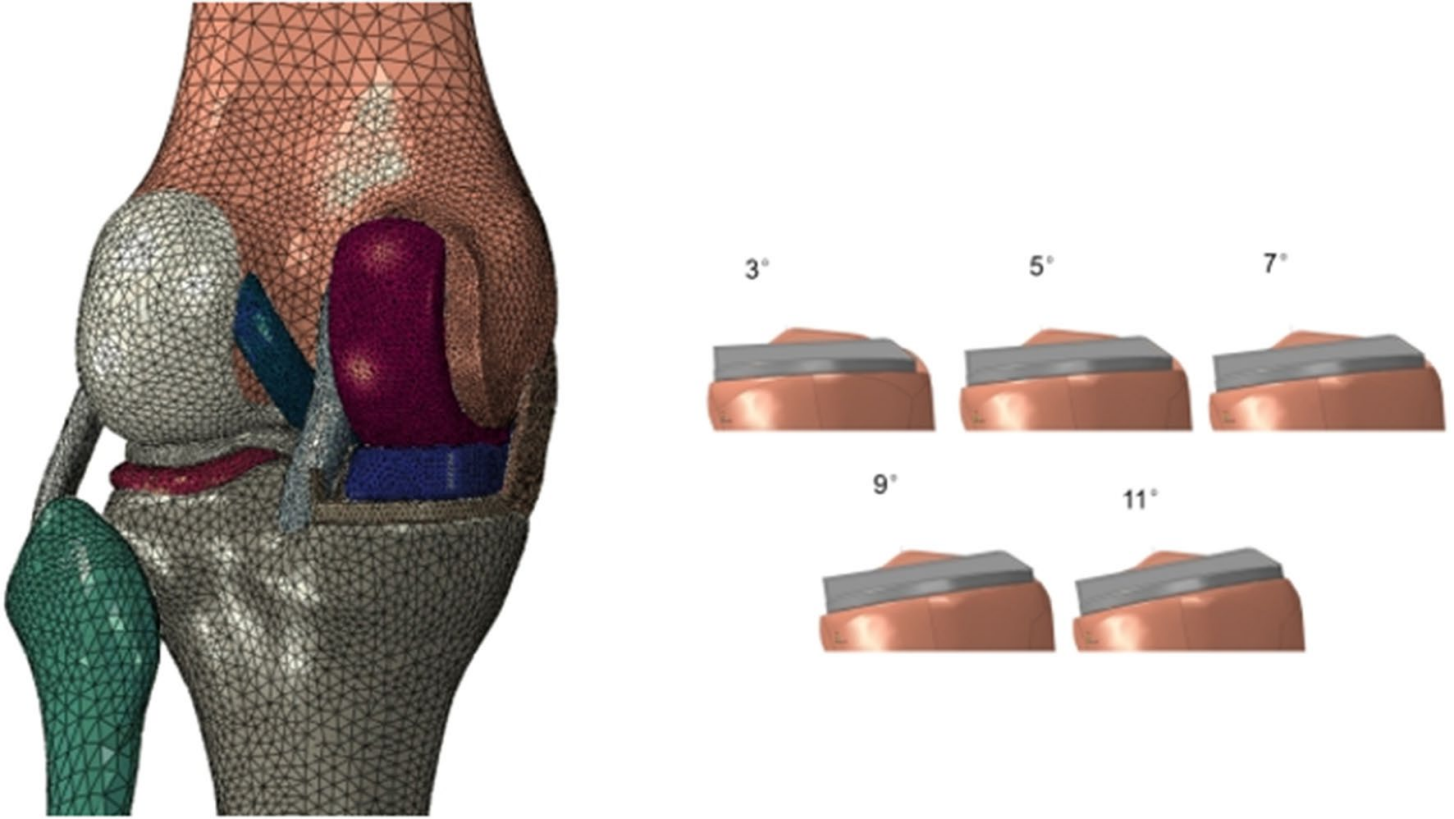
The finite element model of 7°posterior inclination of tibial component and the other different posterior inclinations (3°, 5°, 9° and 11°) of tibial component

3D point cloud data of femoral prosthesis (S), tibial prosthesis (A) and polyethylene insert were obtained using 3D scanner (ARTEC, EVA), and the Rapidform software was used for reverse 3D reconstruction to obtain the geometric model of the prosthesis. On the basis of the above established healthy knee joint, according to the Oxford III generation UKA standard surgical technique, a 7 mm thickness osteotomy was performed with Boolean operation with the 0° inclination in the coronal plane and the 7° posterior inclination in the sagittal plane. On the basis of this model, the tibial prosthesis was rotated to establish 3°, 5°, 9° and 11° posterior inclination models (Fig. 1).

### Material property assignment and mesh

All models were divided by ten-node modified tetrahedral elements. There were 153,727 units of healthy knee joint model. A total of five MB UKA finite element models with different posterior inclinations of tibial prosthesis were established, and each model had about 300,000 units. Cartilage and meniscus were defined as isotropic linear elastic materials [14]. The ligament was defined as an incompressible transversely isotropic hyperelastic material [14], and the Neo Hookean constitutive model was applied. Its constitutive equation is:

$$\Psi = C_{1} \times (I_{1} - 3)$$

where *C*_1_ is the initial shear modulus and* I*_1_ is the first modified invariant of Cauchy Green strain tensor. *C*_1_ values used for the anterior and posterior cruciate ligaments, the medial and lateral collateral ligaments were 6.06, 6.43, 5.83 and 6.06 MPa, respectively. The material parameters of other structures are shown in Table 1.

**Table 1:** Material properties in the finite element models

	Young’s modulus (MPa)	Poisson’s ratio
Bone	17,000	0.3
Cartilage	15	0.46
Meniscus	27.5	0.33
CoCrMo ally	195,000	0.3
UHMWPE	685	0.4

*CoCrMo ally* Cobalt–chromium–molybdenum ally; *UHMWPE* Ultra-high molecular weight polyethylene

### Load and boundary conditions

Healthy knee joint finite element model using frictionless and limited slip surface-to-surface contact relations of the six (contact on location for: room between the medial and lateral cartilage of the femur and tibia, the femoral cartilage and meniscus between surface under the surface, tibial cartilage and meniscus) was made and the calculation made based on penalty function algorithm. MB UKA model had five contacts to set; the medial compartment surface contact which was using a frictionless contact, and the lateral compartment which was using coulomb friction contact, with a friction coefficient of 0.04.

Model verification under axial load: The degree of freedom in flexion and extension direction of femur was fixed, whereas the degree of freedom in other directions of femur was not fixed. While the lower surface of tibia and fibula remained completely fixed, the cartilage and bone as well as ligament and bone were connected in a binding form, and the front and back corners of the meniscus were bound to the tibial plateau. The reference point of femur was defined at the midpoint of the medial chamber and medial epicondyles of femur. An axial load was applied to this reference point (the direction of the load was down the mechanical axis, and the load size was 1000N), and the load distribution of the medial and lateral compartment was calculated and verified by comparison with the literature results [15].

Tibialis anterior drawer under the force model validation: Tibial reference point was defined at the center of the ankle, tibia and fibula coupling constraint main limit degree of freedom completely fixed in the direction of flexion and extension, femoral and tibial reference points put on forward force (the size of the force of 134 N), and the displacement of the tibia compared with literature results [16].

Static standing load model: Model verification was done under constrained coaxial load. A load of 325 N was applied along the mechanical axis of the tibia at the reference point of the femur, with a buckling angle of 0°. The load ratio of the medial and lateral compartment was then calculated.

**Fig. 2:**
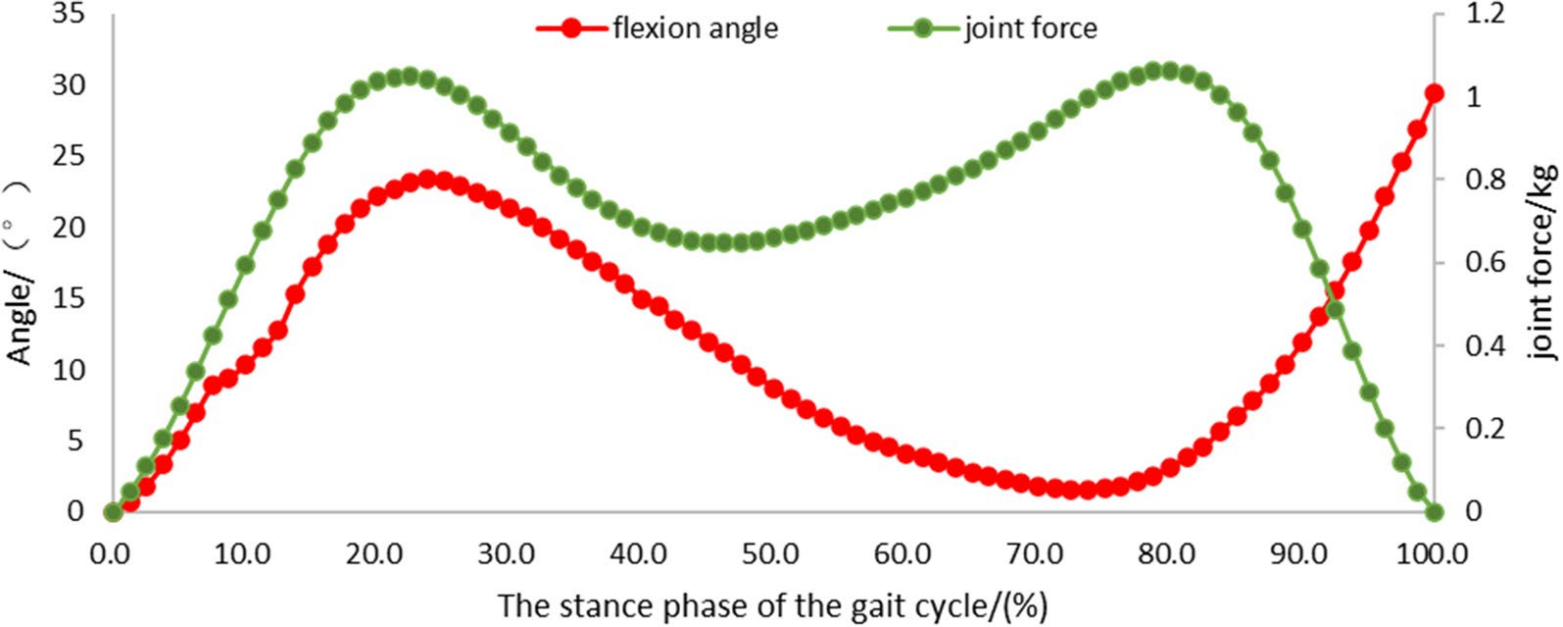
Knee flexion angle and joint force during the stance phase of the gait cycle

Model under gait cycle load: The femur reference point was coupled with the proximal femur, and the model verification done under constrained coaxial load. A force load along the mechanical axis of the tibia and a sagittal angle (flexion and extension) were applied at the femur reference point. The force load and flexion angle were provided by the joint forces and range of motion of the subjects at different points in the gait cycle [17] (Fig. 2). The knee joint force and the angle of flexion and extension at the first peak of ground reaction force and the second peak of ground reaction force in the gait cycle were selected as the loading boundary conditions to calculate the load distribution of the medial and lateral compartments of the knee joint, and the maximum contact pressure between the polyethylene insert and the tibial cartilage of the lateral compartment.

## Results

### Verification of the finite element models of healthy and MB UKA knee joint

**Fig. 3:**
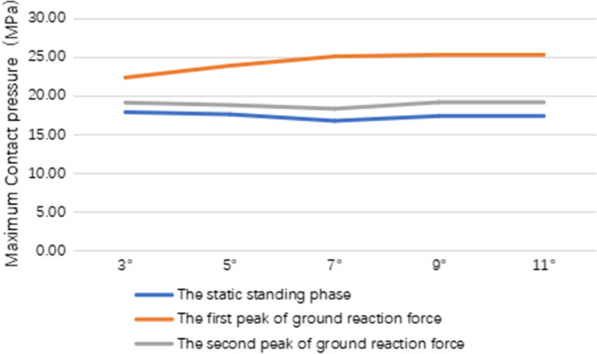
The maximum contact pressures of polyethylene insert

Verification under axial load: In the finite element model of healthy knee joint, the load distribution proportion of the medial compartment accounted for 54.49%, whereas the load distribution proportion of the lateral compartment accounted for 45.51% of the total load, which was consistent with the literature [15] (Fig. 3).

**Fig. 4:**
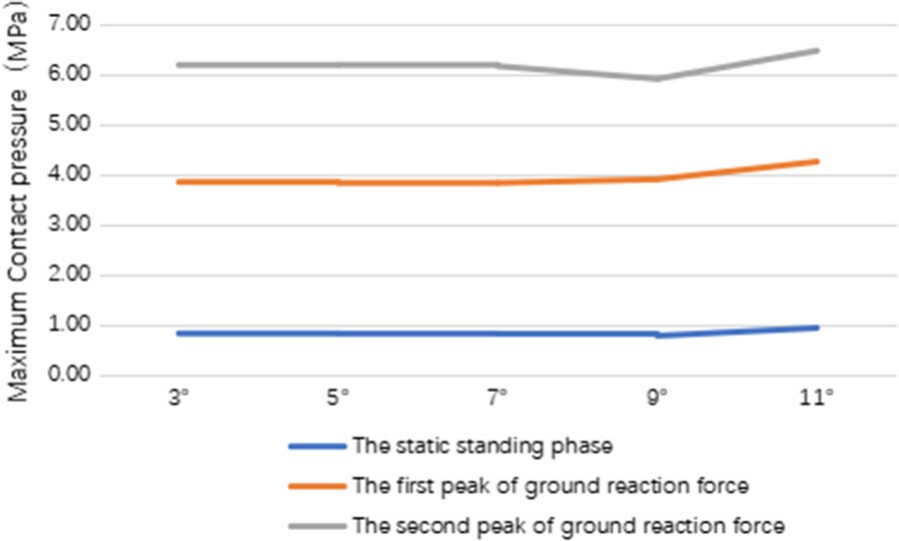
The maximum contact pressures of tibial plateau cartilage in the lateral compartment

The verification under tibialis anterior drawer force: Under the action of 134 N forward and backward drawer force, tibia moved forward 3.89 mm, which was also consistent with literature results [16] (Fig. 4).

**Fig. 5:**
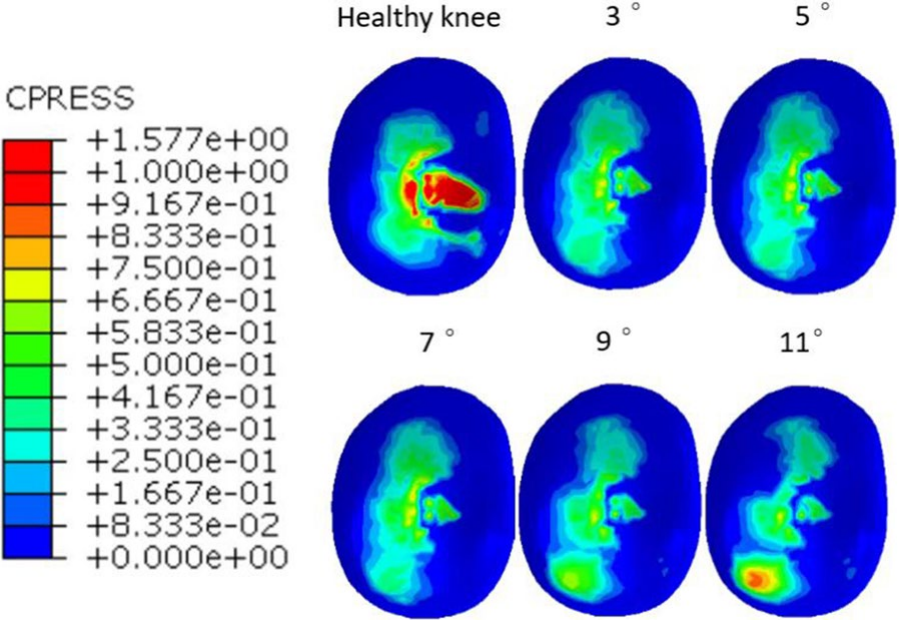
The maximum contact pressures of tibial plateau cartilage in the lateral compartment in the healthy and MB UKA models of different posterior inclinations (3°, 5°, 7°, 9° and 11°) of tibial prosthesis

Comparison of MB UKA and healthy knee finite element model: In the MB UKA model, tilting the of tibial prosthesis 3°, 5°, 7°, 9° and 11° resulted in bearing of 45%, 44.73%, 44.87%, 44.94% and 45.07% of the total load by the medial compartment, respectively. Similarly, the lateral compartment bore 55%, 55.27%, 55.13%, 55.06% and 54.93% of the total load, respectively. The medial and lateral compartments of the healthy knee joint model bore 54.92% and 45.08% of the total load, respectively. The maximum contact pressures of the medial meniscus and lateral tibial plateau cartilage of the healthy knee were 2.14 MPa and 1.57 MPa, respectively. The load distribution proportions of the medial and lateral compartments of the knee after MB UKA were similar to the literature findings [18] (Fig. 5).

### The maximum contact pressures of polyethylene insert

**Fig. 6:**
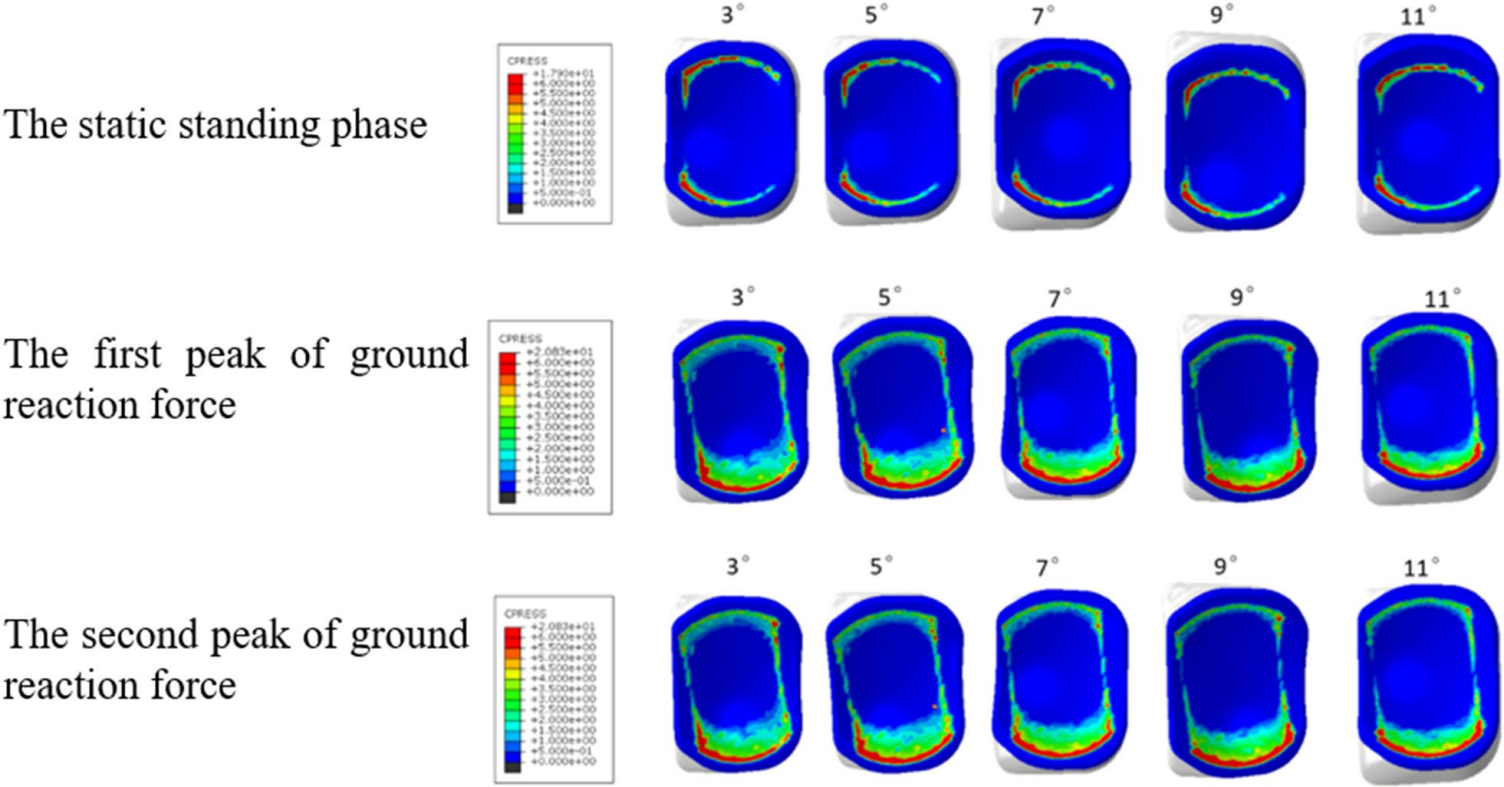
Stress distribution on polyethylene insert

At the static standing phase, the maximum contact pressures of polyethylene insert in MB UKA model with 3°, 5°, 7°, 9° and 11° inclinations were 17.90 MPa, 17.62 MPa, 16.78 MPa, 17.41 MPa and 17.29 MPa, respectively. At the first peak of ground reaction force, the maximum contact pressures of polyethylene insert in MB UKA models with 3°, 5°, 7°, 9° and 11° inclinations were 22.37 MPa, 23.90 MPa, 25.10 MPa, 25.31 MPa and 25.16 MPa, respectively. At the second peak of ground reaction force, the maximum contact pressures of polyethylene insert in MB UKA models with 3°, 5°, 7°, 9° and 11° inclinations were 19.13 MPa, 18.82 MPa, 18.34 MPa, 19.18 MPa and 19.21 MPa, respectively (Fig. 6).

### The maximum contact pressures of tibial plateau cartilage in the lateral compartment

**Fig. 7:**
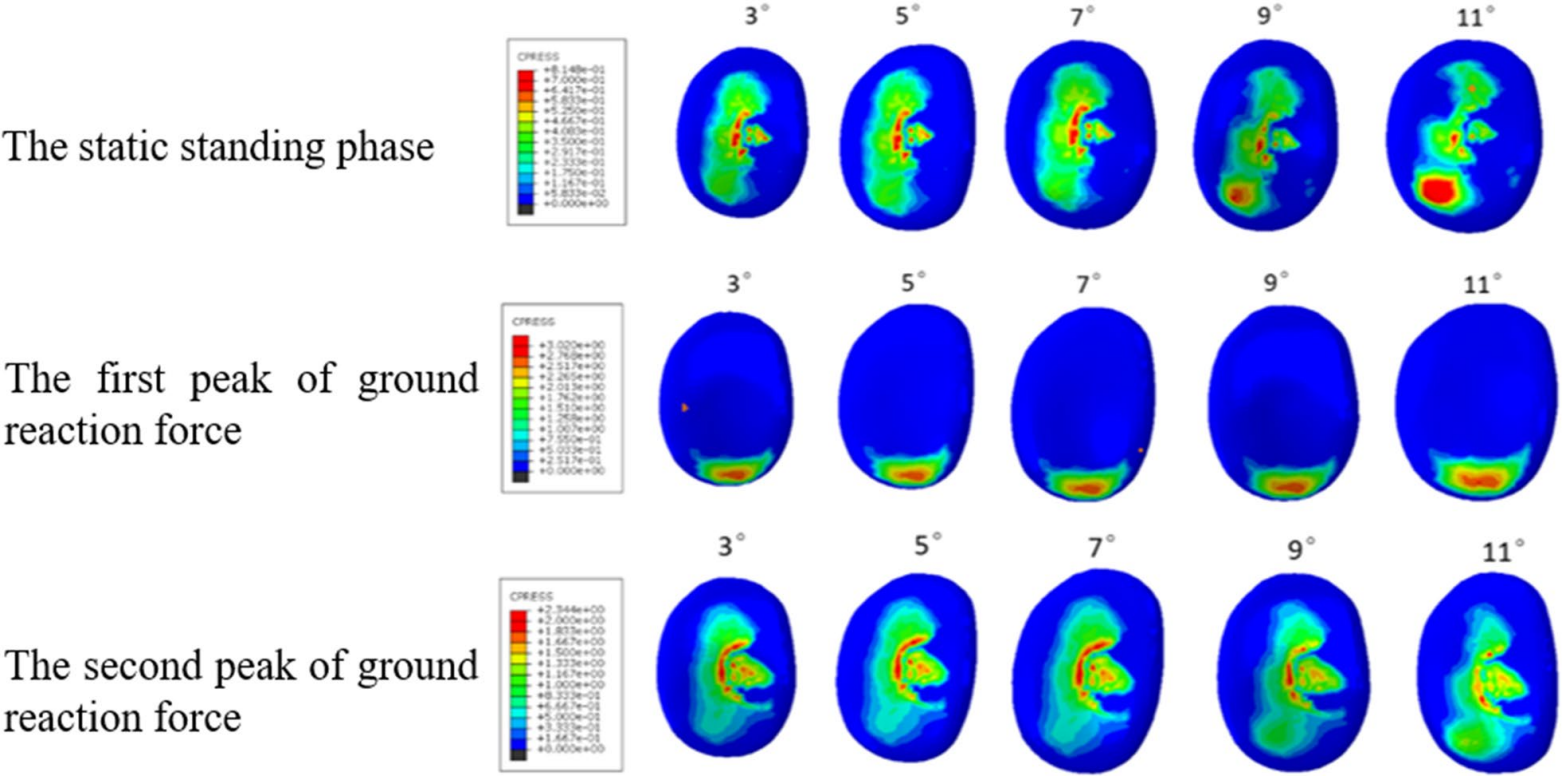
Stress distribution on tibial plateau cartilage in the lateral compartment

At the static standing phase, the maximum contact pressures of tibial plateau cartilage in the lateral compartment were 0.81 MPa, 0.81 MPa, 0.80 MPa, 0.77 MPa and 0.92 MPa, respectively. At the first peak of ground reaction force, the maximum contact pressures of tibial plateau cartilage in the lateral compartment were 3.03 MPa, 3.02 MPa, 3.02 MPa, 3.13 MPa and 3.33 MPa, respectively. At the second peak of ground reaction force, the maximum contact pressures of tibial plateau cartilage in the lateral compartment were 2.34 MPa, 2.36 MPa, 2.34 MPa, 2.01 MPa and 2.22 MPa, respectively (Fig. 7).

## Discussion

Normally, there is a complex biomechanical effect of the posterior inclination on mobile-bearing UKA. The serious problems that arise from malpositioning of the mobile-bearing UKA are progressive ostearthritis, rapid wear on the PE insert and its dislocation, and failure of the ACL [19]. However, to the best of our knowledge, the biomechanical effects of posterior inclination of the tibial prosthesis in mobile-bearing UKA on the knee joint have not been fully elucidated and/or defined through an acceptable research approach. Our results showed that the maximum contact pressures of the polyethylene insert did not change significantly with the increase in the posterior inclination of the tibial prosthesis, while the maximum contact pressures of the tibial plateau cartilage of the lateral compartment increased when the posterior inclination of the tibial prosthesis was > 7°.

In this study, a combination of MRI and CT scans was used to maximize the reconstruction of bone, cartilage, meniscus, and meniscus structures. Due to the complex structure of the model, all the structures adopted a tetrahedral mesh. Within the academic circles, there is still lack of highly recognized process for verification of finite element model. Oberkampf et al. [20], however, advocated four main levels of model verification, namely organizational level, single structure level, multi-structure level and overall level. In the selection of inspection structures, considering that the focus of this study was on load distribution and contact state of the whole knee joint in the gait cycle, the cartilage, meniscus and ligament did not have too much influence on the overall experimental study of the knee joint.

Comparing to the healthy knee model, the MB UKA model was more difficult to verify. Tuncer and Innocenti et al. [21, 22] established the UKA model based on the imaging data obtained from cadaver specimen and carried out matching in vitro tests simultaneously to verify the model. Netter et al. [23] analyzed and compared the data obtained from UKA wear tester and the wear rates obtained from UKA model calculation by using a knee simulation to obtain verification results. Due to the limitation of funds and materials in this study, our finite element model adopted an indirect verification method, and compared the load proportion before and after MB UKA. It further compared the maximum contact stress profile of tibial plateau cartilage in the lateral compartment and polyethylene insert with other studies to verify the validity of the model.

In our study, the proportion of internal and lateral compartment load after MB UKA was consistent with the literature results [18]. Innocenti et al. [18] found that the lateral compartment load of all UKA models was greater than the medial one by studying tibial component alignment from varus 6° to valgus 6°, and similar results were obtained in this study. We believe that the main reason for this result is that the elastic modulus of the prosthesis is much larger than that of the cartilage and meniscus, leading to the redistribution of loads in the knee joint, with a focus on the unreplaced side. Excessive elastic modulus of tibial prosthesis will not only lead to the redistribution of medial and lateral loads, but also change the stress distribution of bone around tibial prosthesis due to the stress shielding effect, which may lead to pain and increase the incidence of aseptic loosening [21].

The greater the maximum contact pressures of the tibial cartilage in the contralateral compartment, the higher the incidence of progressive osteoarthritis in this compartment [24–27]. Studies have shown that the posterior inclination of the tibial prosthesis for MB UKA should not exceed 7°. The tibial inclination of the healthy knee in this study was 7°. Therefore, the 7° inclination of the tibial prosthesis was used as a reference and the original inclination was assumed to be retained. The posterior inclination of tibial prosthetics in normal knee joints has been proven to have a wide range, with an average of 9° [28]. In order to restore the biomechanical environment of the knee as much as possible, the optimal posterior inclination of tibial prosthesis in the sagittal plane of UKA has always been the objective pursued by surgeons. However, the optimal inclination has not been determined clinically and biomechanically. In general, it is recommended that the surgeon restores the patient’s initial tibial posterior inclination after UKA. Our results showed that the maximum contact pressures and pressure concentrations of the tibial plateau cartilage in the lateral compartment were greater when the posterior inclination of the tibial component was excssive (11°).However, these were lesser than the those of a healthy knee joint.

## Conclusions

In this study, finite element models of healthy knee joints including 3D ligament structures were established and verified. Likewise, MB UKA finite element models bearing 5 different posterior inclinations of tibial prostheses (3°, 5°, 7°, 9° and 11°) were established. The results showed that the maximum contact pressures of the polyethylene insert did not change significantly with the increase in the posterior inclination of the tibial prosthesis, while the maximum contact pressures of the tibial plateau cartilage of the lateral compartment increased when the posterior inclination of the tibial prosthesis was > 7°. The stress clouds show that the contact pressures of the polyethylene insert did not change significantly with the increase in the posterior inclination of the tibial component, while the pressure concentrations on the tibial plateau cartilage in the lateral compartment gradually occurred, mainly in the posterior side. The preoperative and postoperative finite element models of MB UKA established in this study reasonably analyzed the contact stress of knee joint, providing a reference method for subsequent MB UKA research. We therefore proposed that excessive osteotomy should be avoided.

## Data Availability

The dataset supporting the conclusions of this article is included within the article. All data are fully available without restriction.

## Abbreviations

UKA: Unicompartmental knee arthroplasty
MB UKA: Mobile-bearing unicompartmental knee arthroplasty
KOA: Knee osteoarthritis
TKA: Total knee arthroplasty
CoCrMo ally: Cobalt–chromium–molybdenum ally
UHMWPE: Ultra-high molecular weight polyethylene
